# Leveraging chatbots for enhanced decision-making: a comprehensive literature review

**DOI:** 10.3389/frai.2026.1748544

**Published:** 2026-04-07

**Authors:** Phiwe M. Simelane, Javeed Kittur

**Affiliations:** 1 Electrical Engineering, Gallogly College of Engineering, University of Oklahoma, Norman, OK, United States; 2 Engineering Pathways, Gallogly College of Engineering, University of Oklahoma, Norman, OK, United States

**Keywords:** chatbot, ChatGPT, decision-making, LLM-based chatbots, systematic literature review

## Abstract

**Introduction:**

Chatbots using large language models such as ChatGPT, Google Bard, etc., have become increasingly popular in recent years. The chatbot’s ability to simulate conversations with users and process input data and respond based on that information has prompted researchers to investigate the applicability of chatbots in decision-making processes across multiple fields. Current literature has investigated the benefits and challenges of using chatbots in a broad context. This paper presents a systematic literature review of current literature discussing chatbots and decision-making processes, exploring the quality of the decision-making process and user perceptions of using chatbots in the decision-making process.

**Methods:**

This SLR aims to provide a comprehensive view of the disciplinary fields in which chatbot decision-making has been evaluated, how chatbots can be used in various fields, and how it compares to human decision-making. Thirty-six articles from seven databases were reviewed in this paper and categorized into six themes: benefits of using chatbot for decision-making, challenges of chatbot-supported decision-making, ethical considerations of using chatbot-supported decision-making, algorithms/tools used in designing chatbots, human vs. AI decision-making, and chatbot decision-making in different fields.

**Results:**

An analysis of these themes revealed (i) benefits of personalized recommendations in decision-making, (ii) issues with inconsistency in output, (iii) ethical concerns about chatbots using sensitive information to make decisions, (iv) ChatGPT’s decision-making is the most studied, and (v) human vs. AI decision-making.

**Discussion:**

The practical and research implications of these findings are further explained in the paper.

## 1 Introduction

In a time of swift technological development and the spread of artificial intelligence (AI), chatbots have become a game-changing tool across a range of industries, changing how businesses communicate with their users and making decisions (Perez-Soler et al., 2018; Reicherts et al., 2022; Huang and Gursoy, 2024; Harris and Kittur, 2024). From basic rule-based systems to complex models driven by natural language processing and machine learning, chatbots—AI-driven conversational agents created to mimic human-like dialogue have undergone significant development (Hussain et al., 2019; Lin et al., 2023; Sbattella, 2023). Their abilities now go beyond responding to inquiries or automating customer support duties; they are becoming more and more important in assisting with intricate decision-making procedures in fields like engineering (Perez-Soler et al., 2018; Alaaeldin et al., 2021), healthcare (Hindelang et al., 2024; Phooriyaphan and Rachsiriwatcharabul, 2025), education (Kooli, 2023; Labadze et al., 2023), and finance (Ramjattan et al., 2021; Mabwe et al., 2025).

### 1.1 Conceptual foundations: chatbots, LLMs, and decision-making

Chatbots are conversational software agents designed to simulate human dialogue through natural language interaction. Early chatbot systems such as ELIZA (Weizenbaum, 1966) relied on rule-based pattern matching and predefined scripts. While these early systems were limited in scope, advances in machine learning and natural language processing (NLP) significantly expanded chatbot capabilities (Wollny et al., 2021).

The development of transformer-based architectures (Vaswani et al., 2017) marked a pivotal shift in language modeling. LLMs trained on massive text corpora, demonstrated the ability to generate contextually coherent responses across diverse domains. Systems such as GPT-3 (Brown et al., 2020) and its successors, including ChatGPT, as well as Google’s Bard, represent a new generation of conversational AI capable of multi-turn dialogue, summarization, reasoning-like outputs, and domain-general assistance. The rapid public adoption of these systems since 2022 has led to widespread integration across education, healthcare, finance, engineering, and business contexts (Dwivedi et al., 2023).

Unlike earlier expert systems that relied on rule-based decision trees, modern LLM-based chatbots are probabilistic models capable of synthesizing information and generating context-aware recommendations, positioning them as potential tools for supporting decision-related tasks.

### 1.2 Decision-making: theoretical context

Decision-making has long been studied within economics, psychology, and management sciences. Classical decision theory distinguishes between normative models, which prescribe how rational decisions should be made, and descriptive models, which explain how decisions are actually made under cognitive constraints (Simon, 1955; Kahneman and Tversky, 2013).

Herbert Simon’s theory of bounded rationality (Simon, 1955) challenged the assumption of perfect rationality by arguing that decision-makers operate under informational, cognitive, and temporal limitations. Later work in judgment and decision-making demonstrated the role of heuristics and biases in shaping human decisions (Tversky and Kahneman, 1974). Contemporary cognitive models further conceptualize decision-making as an information-processing activity involving evaluation of alternatives, outcome prediction, and trade-off analysis (Payne et al., 1993).

In the context of this manuscript, decision-making refers to processes in which alternatives are evaluated and judgments or recommendations are produced based on available data, criteria, and contextual constraints. When mediated by chatbots, these processes involve algorithmic interpretation of user input, retrieval and synthesis of relevant information, and generation of outputs that may influence human judgment.

### 1.3 State of the art: AI and decision support

The integration of computational tools into decision processes is not new. Earlier research on Decision Support Systems (DSS) explored how algorithmic systems could assist human reasoning in managerial and clinical contexts (Power, 2002). However, traditional DSS systems were often structured, rule-based, and domain-specific.

Recent advances in generative AI have expanded this paradigm. Emerging studies have evaluated LLM-based chatbots in clinical triage, financial advisory simulations, educational advising, and managerial contexts (e.g., Ayoub et al., 2023; Levkovich and Elyoseph, 2023; Lakkaraju et al., 2023). These investigations explore both performance accuracy and user perceptions. However, the literature remains fragmented across disciplines, with variability in methodological rigor and evaluation approaches.

To date, there has been limited systematic synthesis focusing specifically on chatbot-supported decision-making across domains. Existing studies often evaluate isolated use cases or single platforms. A comprehensive understanding of how chatbots are being evaluated, where they demonstrate strengths, and where limitations persist remains underdeveloped. This review addresses this gap by synthesizing current empirical and conceptual work on chatbot-mediated decision processes across sectors.

The ability of chatbots to give users context-aware, personalized, and real-time information is what motivates their incorporation into decision-making frameworks (Kim et al., 2020; Shumanov and Johnson, 2021; Liu et al., 2022). Chatbots can lessen cognitive load, improve knowledge access, expedite processes, and promote data-driven insights in settings where decisions must be made quickly (Di Blas et al., 2019; Rooein, 2019). For instance, using patient input and extensive medical databases, chatbots help clinicians diagnose symptoms and suggest treatments in clinical settings (Ben-Shabat et al., 2022; Hirosawa et al., 2023). By identifying patterns, automating data retrieval, and producing useful suggestions, they assist managers in making decisions in business settings (Azmi et al., 2023; Savastano et al., 2024).

The literature is still fragmented, with studies differing greatly in focus, scope, and evaluation metrics, despite the increasing use of chatbots in decision-making contexts. To find the fundamental design principles, evaluate the effects of their use across disciplines, and comprehend the degree to which chatbots improve decision-making, it is imperative to compile the body of existing research. Additionally, user trust, transparency, ethical issues, and chatbot reasoning limitations are still important topics that need careful research. A thorough literature review that summarizes academic research on the application of chatbots to improve decision-making is presented in this paper. This study aims at answering the following research question: “What are the current applications, benefits, and limitations of using chatbots to support decision-making processes in different sectors?”, It seeks to map the state of knowledge today, identify research gaps, and highlight significant trends and technological advancements. By doing this, it provides a comprehensive understanding of how chatbots are changing paradigms for making decisions and suggests lines of inquiry and practice for the future.

## 2 Methods

**Figure 1:**
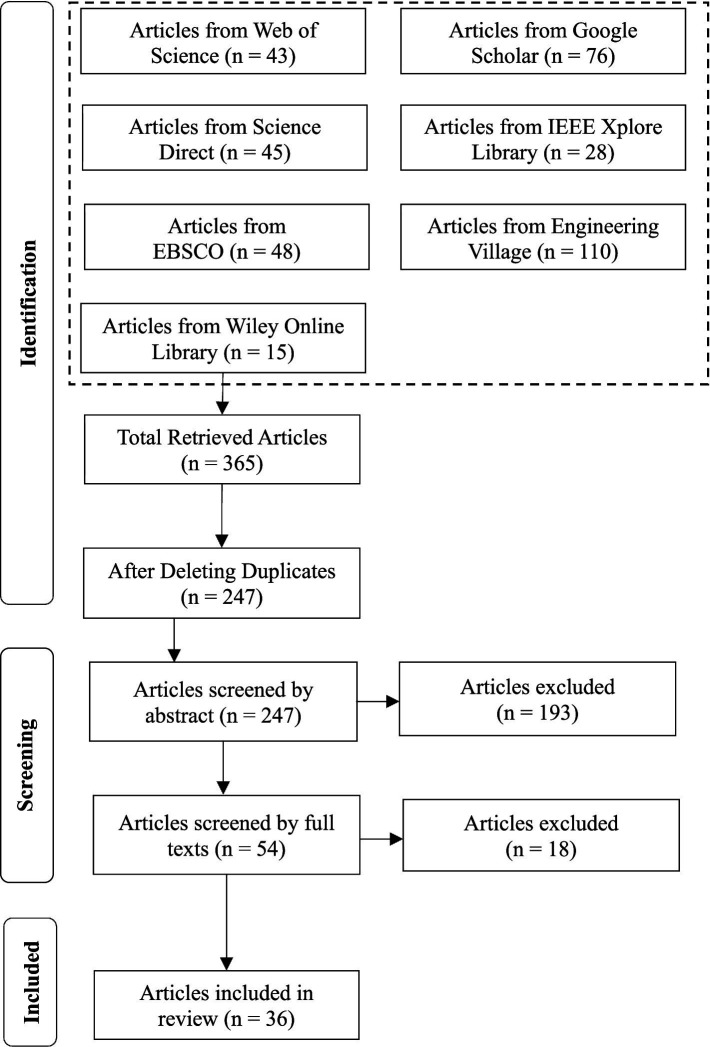
Systematic literature review article selection process using PRISMA.

The Preferred Reporting Items for Systematic Reviews and Meta-Analyses (PRISMA) was selected as the approach we would use to carry out the SLR (Page et al., 2021). This is because this framework is part of the standard approach for SLRs used in different studies (Borrego et al., 2014; Nguyen and Kittur, 2024); it provides structure and ensures our study is a rigorous exploration of the topic. PRISMA is composed of three components: (1) Identification, where relevant articles are gathered from multiple databases using specific search queries; (2) Screening, which involves evaluating articles based on their abstracts and full texts according to predefined exclusion criteria; and (3) Inclusion, where the final set of articles is thoroughly examined to address the research questions. Please refer to Figure 1 for more details on the different phases of the article’s retrieval and selection process. As part of the first phase, we retrieved articles from seven databases: Google Scholar, Web of Science, IEEE explorer, Compendex/Engineering Village, ERIC (Education Resources and Information Center), ScienceDirect, and Wiley Online Library. The articles were selected using the search terms “Chatbots AND Decision Making” and “AI AND Decision Making.” These terms were intentionally selected to maintain a focused scope on chatbot-mediated decision processes rather than broader AI-based decision support systems that do not involve conversational interfaces. Records retrieved from “AI AND decision making” were included only if the abstract/full text indicated an interactive conversational interface (i.e., chatbot or conversational agent) used to support decision-making.

### 2.1 Search date and search string development

Searches were conducted between January 10, 2024, and February 15, 2024, and finalized on Feb 29, 2024. Search strings were developed iteratively through pilot searches to balance sensitivity and conceptual specificity for chatbot-supported decision-making. We used two complementary strings: (1) “chatbot” AND “decision making”* to capture literature explicitly framing conversational systems as chatbots, and (2) “AI” AND “decision making” to capture studies that evaluate conversational AI decision support but may use alternative umbrella terms (e.g., generative AI, large language models, conversational AI) rather than “chatbot.” Records retrieved under the AI-based string were retained only if screening confirmed a conversational agent/chatbot component aligned with the study’s scope.

### 2.2 Operational constraints and record exports

Database interfaces often report very large approximate “hit counts,” especially in Google Scholar, which are not equivalent to a reproducible set of retrievable records. Accordingly, consistent with reproducible review practice, we report the number of records exported for screening under explicitly defined operational constraints rather than the raw hit counts. For Google Scholar, we applied: (i) publication year filter 2014–2024, (ii) exclusion of patents, books, book chapters, editorials, magazines, and citations, (iii) sorting by relevance, and (iv) exporting all relevant results per search string (the relevance was determined by manual screening). The exported records from each database were then deduplicated and screened per PRISMA. Thus, the PRISMA “Articles from Google Scholar (*n* = 76)” reflects the number of articles that were included in the pool and screened, not the database-reported hit count.

Table 1 provides the database search results and screening summary across the sources. The “Search Results (before deduplication)” column represents the total number of records returned by each database using the defined search strings and filters. Due to the large volume of retrieved records, an initial title-based screening was conducted to identify studies directly relevant to chatbot-supported decision-making, resulting in the “Articles After Initial Title Review.” The “Retrieved Articles in the Pool” column represents the subset of articles retained after removing clearly irrelevant records and preparing them for abstract and full-text screening. These records were subsequently deduplicated and evaluated according to the PRISMA screening process, leading to the final set of included studies.

**Table 1:** Database search results and screening summary across sources.

Database	Search fields	Filters applied	Search Results (before deduplication)	Articles after initial title review	Retrieved articles in the pool
Web of Science	Topic (title, abstract, keywords)	Years: 2014–2024; English; document types: articles & reviews	*n* = 867	*n* = 56	*n* = 43
Google Scholar	Title, abstract, keywords (where available)	Years: 2014–2024; English; patents and citations excluded	*n* = 35,364	*n* = 105	*n* = 76
ScienceDirect	Title, abstract, keywords	Years: 2014–2024; Research articles; English	*n* = 502	*n* = 53	*n* = 45
IEEE Xplore	Metadata (title, abstract, index terms)	Years: 2014–2024; Journals & Conferences; English	*n* = 653	*n* = 44	*n* = 28
ERIC (EBSCO)	Title & abstract	Years: 2014–2024; Peer-reviewed; English	*n* = 209	*n* = 68	*n* = 48
Engineering Village (compendex)	Title & abstract	Years: 2014–2024; English	*n* = 29,356	*n* = 136	*n* = 110
Wiley Online Library	Title & abstract	Years: 2014–2024; English	*n* = 20,509	*n* = 42	*n* = 15

Database-reported hit counts (particularly in Google Scholar) often reflect approximate retrieval estimates rather than reproducible record sets. Therefore, consistent with systematic review best practices, we report the number of records exported and screened under explicitly defined filters and operational constraints rather than the total database-reported hit count.

### 2.3 Data collection

The titles of the 365 articles retrieved were saved in an Excel file. The article titles across different databases were compared to remove the duplicate articles. These articles were screened by abstract against the following four exclusion criteria: EC1: Articles published before 2014 will be excluded, EC2: Articles not published in English will be excluded, EC3: Articles that do not focus on decision making using chatbots will be excluded; EC4: Articles that are work-in-progress or short papers will be excluded. The remaining articles were screened by full text against the same criteria. Out of 365 retrieved articles, 329 articles were excluded: 118 were duplicates, 193 by abstract, and 18 by full text (please refer to Figure 1).

A final set of 36 articles were then reviewed in detail and different information from these articles was saved in an Excel file. The information included the year, country of first author, research question, methodology, data collected, population and sample size, sampling methods, data analysis techniques, findings, implications, limitations and future work were gathered from each paper. These summaries were retrieved and sorted with the help of NotebookLM (Google, 2024) and verified manually after retrieval.

### 2.4 Data analysis

The final set of included articles was analyzed using a structured thematic synthesis approach. Relevant information was extracted from each study, including application domain, research objectives, study design, evaluation method, and key findings related to chatbot-supported decision-making. An inductive coding process was used to identify recurring patterns and concepts across studies. During the initial coding phase, each article was reviewed and assigned descriptive codes representing key ideas, methodological approaches, and reported outcomes. These codes were derived directly from the study content rather than from a predefined coding framework. The codes generated include user friendly, inconsistencies and inaccuracies, financial decision-making, and data privacy concerns. In the subsequent phase, related codes were grouped into broader thematic categories based on conceptual similarity. This process involved iterative comparison and refinement to ensure that themes accurately reflected patterns observed across multiple studies. Themes were refined until clear distinctions emerged between categories and no new themes were identified. The resulting themes formed the basis for the thematic synthesis presented in the Results section.

#### 2.4.1 Methodological strengths and limitations

This review followed a structured and transparent methodology guided by the Preferred Reporting Items for Systematic Reviews and Meta-Analyses (PRISMA) framework to ensure consistency and reproducibility in study identification, screening, and selection. Multiple academic databases spanning engineering, education, healthcare, and interdisciplinary domains were included to capture a broad and representative sample of relevant literature. The use of clearly defined inclusion criteria, documented screening procedures, and systematic thematic synthesis further strengthened methodological transparency and analytical consistency.

Several methodological limitations should also be acknowledged. First, although multiple databases were searched, relevant studies indexed in other databases or published outside the selected sources may not have been captured. Second, the search strategy relied on conceptually focused search strings designed to identify chatbot-supported decision-making studies; however, terminology in this rapidly evolving field varies across disciplines, and some relevant studies may use alternative descriptors. Although only two primary search strings were used, they were intentionally selected to preserve conceptual clarity and focus on chatbot-mediated decision-making. We acknowledge that adjacent terminology (e.g., conversational agents or decision support systems) may be used in some disciplines; however, expanding the search to these broader categories could introduce systems that do not align with the conversational AI focus of this review. Third, due to the rapid pace of advancement in generative AI technologies, the included literature reflects the state of research available at the time the search was conducted and finalized. As data collected was stopped in Feb 2025, the most recent included studies were approximately 1–1.5 years old at the time of submission. Given the fast-evolving nature of chatbot and large language model technologies, newer developments and evaluations may not be fully represented in this review. Finally, while structured coding and thematic synthesis were applied to ensure systematic analysis, thematic interpretation inherently involves analytical judgment (Paul and Barari, 2022).

#### 2.4.2 Assessment of methodological rigor

Due to the multidisciplinary and methodologically heterogeneous nature of the included studies, a formal risk-of-bias assessment tool was not applied. The 36 articles encompassed a wide range of designs, including case-based evaluations, cross-sectional surveys, quasi-experiments, controlled laboratory studies, conceptual analyses, system development papers, and prior literature reviews (see Table 2).

**Table 2:** Disciplinary fields explored.

#	Disciplinary field	References
1	Medical field	Saban and Dubovi (2024), Kumar and Joshi (2022), Garcia Valencia et al. (2023), Dronkers et al. (2024), Mihalache et al. (2024), Huo et al. (2024), Ayoub et al. (2023), Levkovich and Elyoseph (2023), Sarangi et al. (2024), Rodriguez-Arrastia et al. (2022), and Bužančić et al. (2023).
2	Finances/managerial	Kumari et al. (2023), Reicherts et al. (2022), Ferreira et al. (2021), Lakkaraju et al. (2023), Azmi et al. (2023), Temara et al. (2024), Jhaveri et al. (2023), Ramjattan et al. (2021), Wang and Wu (2024), Morana et al. (2020), Alaaeldin et al. (2021), and Palade and Ion (2022).
3	Academic advising	Deshpande et al. (2024), Hsu et al. (2023), Cloux and Monticolo (2023), Karyotaki et al. (2022), and Aciang Iku-Silan et al. (2023).
4	Software testing	Perez-Soler et al. (2018) and Gomes (2024).
5	General or miscellaneous	Leschanowsky et al. (2023), Sharman (2024), Manivannan et al. (2023), Ingrams et al. (2021), and Gurkan and Yan (2023).

A notable proportion of studies relied on hypothetical scenarios or vignettes to evaluate chatbot decision-making performance, which may limit ecological validity and generalizability. Several studies used self-reported user perception measures, introducing potential response bias. Additionally, sample sizes varied considerably, and some development-focused papers lacked external validation against real-world datasets.

Conversely, a subset of studies incorporated comparative evaluations against human experts, statistical accuracy metrics, or controlled experimental conditions, demonstrating higher methodological rigor. Given this variability, findings from this review should be interpreted with consideration of differences in study design, validation approaches, and disciplinary standards.

#### 3 Findings

We begin by providing a comprehensive assessment of research and practice trends in Chatbot decision-making articles published between 2014 and 2024. We present descriptions, exemplary studies, research and practical implications for the six themes discovered after synthesizing 36 publications.

##### 3.1 Descriptive findings related to publication trends

###### 3.1.1 Publication per year

**Figure 2:**
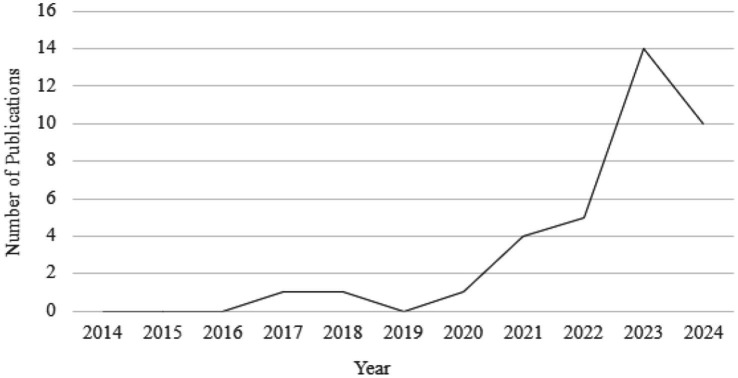
Number of publications in chatbot decision-making by year.

Figure 2 shows there has been an increase in the number of articles about chatbot decision-making per year, as there were no papers published from 2014 to 2016, then an exponential increase in papers released from 2021 to 2024, reaching a high of 14 and 10 papers in 2023 and 2024. It is important to note that the data collected was stopped on Feb 29, 2024, and hence the number of articles for the year 2024 are not fully accounted for/considered. This positive trend is indicative of the growing popularity of AI technology and suggests an increasing number of researchers are investigating ways in which this technology can be used. This upward trend is also indicative of the increase in investment into exploring the potential use of Chatbots in decision-making by identifying ways chatbots can be used effectively to make decisions and areas that need to be improved for the technology to be used as a decision-making tool.

###### 3.1.2 Country of affiliation of first author

The first authors from the selected articles were from eighteen different countries. Most of the authors were from the United States (US) (16.6%) and India (16.6%), and the remaining papers were more or less evenly distributed among sixteen countries. The implementation of an exclusion criteria that removed non-English papers influenced the likelihood of selected papers originating from certain countries and consequently, the countries of affiliation for the first author. This is demonstrated by the fact that countries with the most author affiliations, the US and India, had English as one of the official languages. Table 3 shows the countries of affiliation of the authors of the selected articles.

**Table 3:** Distribution of authors by country of affiliation.

Country of affiliation	*N*	%
India	6	16.6
USA	6	16.6
Canada	2	5.5
Israel	2	5.5
Morocco	2	5.5
Spain	2	5.5
Taiwan	2	5.5
UK	2	5.5
China	1	2.7
Croatia	1	2.7
Egypt	1	2.7
France	1	2.7
Greece	1	2.7
Nepal	1	2.7
Norway	1	2.7
Portugal	1	2.7
Romania	1	2.7
Sweden	1	2.7
The Netherlands	1	2.7
Trinidad and Tobago	1	2.7

###### 3.1.3 Areas of exploration

Table 2 shows the chatbot decision-making investigated across various fields among the sampled articles, including the medical field, finances, and education. The finance field, which included papers discussing corporate, managerial, and marketing decision-making, made up the majority of the selected articles (33.3%), closely followed by clinical decision-making (30.5%) and education (13.8%). A few articles explored group decision-making (5.5%), software-related topics (2.7%), and perceptions in government decision-making (2.7%). The rest of the articles covered a broad range of topics, including ethics and user perceptions, which could not be categorized under any disciplinary field.

###### 3.1.4 Methods of investigation of chatbot decision-making

The articles selected for this study were grouped by the research methodology they used to investigate the quality of chatbot decision-making (please refer to Table 4). In total, there were 11 approaches implemented, with the majority (72.72%) using primary research methods and the rest (27.27%) using secondary research methods. Overall, most of the studies used vignettes and created hypothetical cases or used case studies from current literature to evaluate chatbot decision-making with regards to those cases. The next most popular method of investigation was the design of a custom chatbot using a decision tree, Google Dialog Flow, and other natural language processing (NLP) algorithms to assess its decision-making performance in specific situations and disciplinary fields.

**Table 4:** Methodology employed in articles.

#	Methods of investigation	References
1	Case-based/hypothetical scenarios/vignette	Banerjee et al. (2017), Ingrams et al. (2021), Reicherts et al. (2022), Saban and Dubovi (2024), Lakkaraju et al. (2023), Kumar and Joshi (2022), Sharman (2024), Dronkers et al. (2024), Mihalache et al. (2024), Huo et al. (2024), Ayoub et al. (2023), Levkovich and Elyoseph (2023), Manivannan et al. (2023), and Bužančić et al. (2023).
2	Cross-sectional design	Saban and Dubovi (2024) and Sarangi et al. (2024).
3	Quasi experimental	Hsu et al. (2023) and Aciang Iku-Silan et al. (2023).
4	Designed chatbots	Reicherts et al. (2022), Ferreira et al. (2021), Perez-Soler et al. (2018), Gurkan and Yan (2023), Ramjattan et al. (2021), Karyotaki et al. (2022), and Alaaeldin et al. (2021).
5	Using general chatbot	Deshpande et al. (2024), Cloux and Monticolo (2023), Wang and Wu (2024), and Rodriguez-Arrastia et al. (2022)
6	Comparative methodology	Gomes (2024)
7	Qualitative methodology	Leschanowsky et al. (2023)
8	Controlled laboratory experiment	Morana et al. (2020)
9	Literature review	Kumari et al. (2023), Garcia Valencia et al. (2023), and Palade and Ion (2022)
10	Conceptual and analytical approach/examples	Azmi et al. (2023) and Temara et al. (2024)
11	Exploratory approach	Jhaveri et al. (2023)

Although a significant number of the literature on this subject tested chatbots and decision-making algorithms using case studies and vignettes, a number of authors noted some of the limitations. Firstly, due to the use of hypothetical scenarios that were made up or derived from current literature in the disciplinary field, authors noted that the scenarios the chatbots were tested on were very specific, and thus the findings of those studies could not be generalized to the broader disciplinary field (Banerjee et al., 2017; Ayoub et al., 2023; Ingrams et al., 2021; Reicherts et al., 2022; Saban and Dubovi, 2024; Dronkers et al., 2024; Mihalache et al., 2024).

###### 3.1.5 Methods of evaluation of chatbot decision-making quality

Table 5 summarizes the methods that were utilized by the researchers to assess the quality of the decisions made by chatbots. Most of the articles (*N* = 12) collected primary data from participants using qualitative methods such as interviews, surveys, open-ended questions; and focus groups; and quantitative methods including questionnaires and multiple-choice questions. The next most common method of evaluation was a direct comparison of chatbot output to experts in a disciplinary field (*N* = 4) and statistical analysis on the accuracy of the chatbot output (*N* = 4).

**Table 5:** Methods used to evaluate the quality of Chatbot decision-making.

#	Methods of evaluation	References
1	MCQ/surveys/interviews/open-ended questions	Banerjee et al. (2017), Ingrams et al. (2021), Reicherts et al. (2022), Gurkan and Yan (2023), Leschanowsky et al. (2023), Cloux and Monticolo (2023), Ramjattan et al. (2021), Morana et al. (2020), Alaaeldin et al. (2021), Aciang Iku-Silan et al. (2023), Rodriguez-Arrastia et al. (2022), and Manivannan et al. (2023).
2	Chatbot-assisted vs. traditional decision-making	Ferreira et al. (2021), Perez-Soler et al. (2018), Gomes (2024).
3	Comparisons between experts and output	Saban and Dubovi (2024), Wang and Wu (2024), Dronkers et al. (2024), Levkovich and Elyoseph (2023), Huo et al. (2024), Ayoub et al. (2023), and Sarangi et al. (2024).
4	Analysis of current literature and case studies	Azmi et al. (2023), Palade and Ion (2022), Kumar and Joshi (2022), Garcia Valencia et al. (2023), Karyotaki et al. (2022), Temara et al. (2024), Jhaveri et al. (2023), Sharman (2024), Kumari et al. (2023), and Hsu et al. (2023).
5	Evaluation of output	Deshpande et al. (2024), Lakkaraju et al. (2023), Mihalache et al. (2024).
6	Statistical analysis/standard deviation	Gurkan and Yan (2023), Ayoub et al. (2023), Levkovich and Elyoseph (2023), and Sarangi et al. (2024).

Collecting primary data using methods such as interviews and quantitative and qualitative surveys was the preferred method of evaluation due to its ability to provide researchers with user perceptions of the technology and the quality of decision-making (Gurkan and Yan, 2023). However, Ramjattan et al. (2021) highlighted that the reliance on self-reported data introduces the possibility of bias from participants in their perceptions in favor or against chatbots in decision-making.

###### 3.1.6 Algorithms used

The different chatbots, algorithms, and decision-making technologies that were investigated by the sampled articles were grouped together are shown in Table 6. Of the remaining studies that investigated technology, 11 studies did not specify the types of generative AI or large language model chatbots they used in their study. Among the studies that identified the technology examined, ChatGPT 3.5–4.0 was the most researched chatbot, with 12 studies evaluating its use across various disciplines. The second most studied were custom chatbots created by researchers using unspecified NLPs, with five studies focusing on them.

**Table 6:** Algorithms used in the sampled articles.

#	Algorithms used	References
1	Generative AI	Morana et al. (2020), Garcia Valencia et al. (2023), Sharman (2024), Jhaveri et al. (2023), Temara et al. (2024), Azmi et al. (2023), Kumar and Joshi (2022), Kumari et al. (2023), Ingrams et al. (2021), Banerjee et al. (2017), and Cloux and Monticolo (2023).
2	Decision-making tree	Aciang Iku-Silan et al. (2023) and Deshpande et al. (2024).
3	KNN	Deshpande et al. (2024).
4	Adaboost classifier	Deshpande et al. (2024).
5	Naive bayes	Deshpande et al. (2024).
6	ChatGPT 3.5/4.0	Sarangi et al. (2024), Levkovich and Elyoseph (2023), Ayoub et al. (2023), Huo et al. (2024), Mihalache et al. (2024), Dronkers et al. (2024), Wang and Wu (2024), Leschanowsky et al. (2023), Lakkaraju et al. (2023), Gomes (2024), Saban and Dubovi (2024), and Bužančić et al. (2023).
7	Expert decision making based chatbot	Hsu et al. (2023).
8	Conventional Chatbot	Hsu et al. (2023).
9	Custom-made chatbots	Alaaeldin et al. (2021), Karyotaki et al. (2022), Gurkan and Yan (2023), Perez-Soler et al. (2018), and Reicherts et al. (2022).
10	Bard	Huo et al. (2024), Lakkaraju et al. (2023), and Gomes (2024).
11	Copilot	Huo et al. (2024) and Gomes (2024).
12	Blenderbot	Leschanowsky et al. (2023).
13	Chatbot developed with google dialog	Ramjattan et al. (2021) and Ferreira et al. (2021).
14	Bingchat	Sarangi et al. (2024).
15	Llama 2.0	Dronkers et al. (2024).
16	IBM Watson assistant	Aciang Iku-Silan et al. (2023).
17	Perplexity	Sarangi et al. (2024), and Huo et al. (2024).
18	Claude	Sarangi et al. (2024).

##### 3.2 Thematic analysis: descriptions, exemplars, and implications

In this section, the previously identified themes (benefits of chatbot decision-making, challenges of chatbot decision-making, ethical considerations of using chatbot-supported decision-making, Algorithms/tools used in designing chatbots, Human vs. AI decision-making, and chatbot decision-making in different fields) are detailed. This is done by introducing the theme, an overview of the articles that discuss findings related to the theme, identifying and presenting two exemplar studies with the strongest connection to the theme, and lastly discussing the research and practical implications for each theme. Table 7 identifies each of the six themes, the codes mapped to that theme (see Appendix A for code definitions), and the number of articles categorized under that theme. Appendix B is a table mapping the articles to the themes they discuss, some of which discussed multiple themes. Although themes were categorized discretely for analytical clarity, conceptual overlaps emerged, particularly between technological limitations and comparative performance analyses.

**Table 7:** Distribution of sampled articles by thematic classification.

Theme	Description	Codes	*N*
Benefits of using chatbot in decision-making	This theme is an overview of how chatbots’ ability to simplify complex data, make personalized recommendations, and use a data-driven approach can be utilized to enhance decision-making processes.	Improves group decision-making Not susceptible to bias like humans Relieving humans of mundane tasks saves time Personalized recommendations Data-centric approach User-friendly Simplification of complex data Fast response time Reduce barriers for participation	19
Challenges of chatbot-supported decision-making	This theme encompasses the broad drawbacks of using chatbots as decision-making tools. These drawbacks were noted in a majority of the research papers and include their inconsistency and inaccuracies. Which lead users to make incorrect decisions, thus undermining humans’ decision-making.	Inaccurate and dangerous suggestions Inconsistent output Users not understanding feedback from chatbot Limited language range Output relies on quality of input data	17
Ethical consideration of using chatbot-supported decision-making	This theme encompasses topics related to concerns about the privacy of the user’s data and the idea of regulations and guidelines being implemented when using chatbot for financial and medical decision making in particular,	Data privacy concerns Ethical and privacy regulations and guidelines required Manipulation of user for information	7
Algorithms/tools used in designing chatbots	This theme includes topics related to the different types of algorithms and chatbots used in the decision-making process. It summarizes how well they performed in said process.	Perplexity for open-ended questions Decision tree ChatGPT in clinical decision-making ChatGPT can be used as a Multi-Criteria Decision Maker Custom-made chatbots	8
Human vs. AI decision-making	This theme explores how chatbot-supported decision-making compares to human decision-making. It outlines the shortcomings of technology and how it mirrors and differs from human decision-making.	Not susceptible to bias like humans Make decisions by the book High rates of intelligence decay Did not match human expert performance	5
Chatbot decision-making in different fields	This theme encompasses all discussions related to the decision-making by chatbots and/or algorithms in different areas of knowledge, including medical decisions for various medical procedures, financial advice for both business owners and individual investors, and academic advising for students.	Inaccurate and dangerous suggestions Group decision-making Clinical decision-making Financial decision-making Mathematical errors Decision tree is more accurate Personalized recommendations Guiding student career choice	24

**Figure 3:**
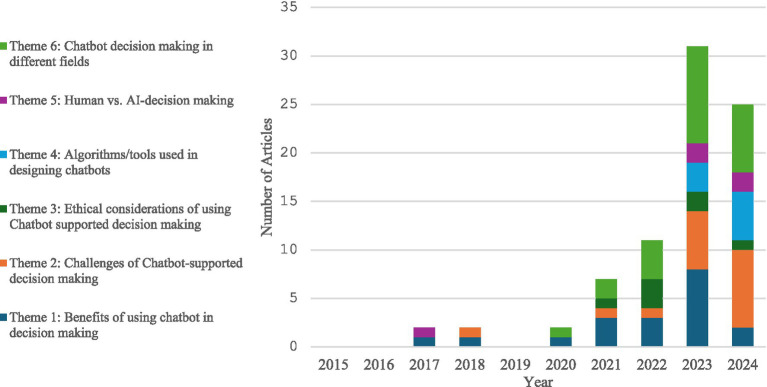
Frequency of occurrences of themes across the house.

Figure 3 shows the frequency at which each theme was discussed from 2015 to 2024. Theme 1: chatbot decision-making is the theme that has been discussed the most over the years, particularly in 2023. It was followed by papers exploring the challenges of chatbots in decision-making. Furthermore, the explorations into the algorithms and tools used to make chatbots for decision-making were first published in 2023 and have increased since.

##### 3.2.1 Theme 1: benefits of using chatbot for decision-making

A total of 19 articles were categorized under the benefits of using chatbots for decision-making. The selected articles highlight the potential for chatbots having a significant and positive impact on decision-making processes in general. These articles can be further subdivided into their user-friendly aspects, which outline how technology’s ability to consistently produce positive interactions with users by providing them with personalized recommendations and simplification of complex data can be used to enhance decision-making (Ingrams et al., 2021; Perez-Soler et al., 2018; Kumar and Joshi, 2022; Ferreira et al., 2021; Reicherts et al., 2022; Temara et al., 2024; Cloux and Monticolo, 2023; Garcia Valencia et al., 2023; Morana et al., 2020; Karyotaki et al., 2022; Aciang et al., 2023; Azmi et al., 2023). The second subsection of this theme would be how chatbots handle data. This subtheme encompasses its fast data processing and response times, its data-centric approach, etc. (Jhaveri et al., 2023; Alaaeldin et al., 2021; Manivannan et al., 2023; Levkovich and Elyoseph, 2023; Gomes, 2024).

###### 3.2.1.1 Exemplar study 1

This exemplar was chosen because it highlights a significant number of the benefits that can come with using AI chatbots in the decision-making process. This exemplar by Jhaveri et al. (2023) demonstrates the positive impact of taking advantage of chatbots relying on data and consequently making data-driven decisions when it comes to marketing. This paper used secondary data to investigate the use of AI chatbots in marketing and concluded that AI analyzes customer needs and purchasing behavior, which allows marketers to find profitable consumer segments, target these consumer groups, and improve product creation and advertising. This data-centric approach improves the marketers’ interactions with potential customers, which has the potential to lead to more productive advertising methods and increase the revenue generated by businesses that implement the technology.

###### 3.2.1.2 Exemplar study 2

Another exemplar by Levkovich and Elyoseph (2023) captures how chatbots can enhance clinical decision-making when evaluating and treating patients’ depressive episodes. This study investigated the performance of ChatGPT 3.5 and ChatGPT 4.0 compared to physicians in the treatment of patients suffering from depression. For mild depression ChatGPT 3.5 and 4.0 recommended psychotherapy in 95 and 97.5% of the case studies, respectively, a treatment in line with the guidelines for managing depression, compared to 48.3% of physicians. Further, for severe depression, ChatGPT-3.5 and ChatGPT-4 suggested psychotherapy and pharmacological treatment’ in 72 and 100% of cases, which is in line with guidelines, while 44% of French physicians suggested the same treatment plan. The chatbots were also found to exhibit no significant differences in therapeutic approach between blue-collar and white-collar workers (*p* = 0.49 and *p* = 0.82 for ChatGPT-3.5 and ChatGPT-4, respectively), unlike physicians who, according to Dumesnil et al., tend to combine psychotherapy and prescriptions for upper-class workers but only prescription medication for lower-class workers. These results demonstrate that technology can make decisions in line with recommended treatment guidelines and not be influenced by the socioeconomic status and gender of patients. Authors noted that ChatGPT has the potential to improve clinical decision-making in mild depression while promoting equity in treatment, particularly as physicians can face external factors such as a high number of patients, limited time and a large variety of mental illnesses.

###### 3.2.1.3 Research implications

These findings of the articles categorized under this theme present several benefits that could be further explored in future research papers for healthcare, business, and other fields. Future studies should explore the application of various AI language models to assess the benefits of the chatbots’ adaptability and accuracy across different fields and stages of decision-making. In addition, further research is needed to understand how chatbot assistance leads to improvements in decision-making, for example, by examining factors like communication patterns and bias mitigation within team decision-making (Gurkan and Yan, 2023). Investigating the factors that make chatbots beneficial could be crucial in finding ways to maximize the positive impact of chatbots in decision-making across the board (Gurkan and Yan, 2023). Investigating conversational feedback loops and iterative test prioritization could enhance human-AI collaboration, leading to more informed decision-making and optimized testing outcomes (Gomes, 2024).

###### 3.2.1.4 Practice implications

Chatbot’s ability to use high-quality input data to develop personalized recommendations gives it the potential to be used as a tool by underserved regions to address healthcare disparities, particularly through the development of telemedicine platforms and mobile health applications (Kumar and Joshi, 2022). Furthermore, chatbots have demonstrated high levels of efficiency with time, providing quick responses and simplifying complex tasks, thus opening the door for technology to be leveraged in areas that require the efficient use of time, which can free up time for more serious decision-making, consequently enhancing the decision-making process. For example, chatbots can be utilized for scheduling and project tracking for managers as well as financial management, which could reduce time spent on operations by 60% (Ferreira et al., 2021). Given that chatbots in team decision-making were shown to create a positive relationship between team decision-making outcomes and early chatbot assistance, with a *p*-value less than 0.05, this suggests that chatbots in team decision-making process, especially at the beginning, can be beneficial and produce better outcomes (Gurkan and Yan, 2023). Lastly, the literature examined under this theme highlighted chatbots’ lack of susceptibility to human biases and their potential to be used in collaboration with humans to arrive at more robust decisions and mitigate human limitations and biases (Gomes, 2024; Gurkan and Yan, 2023).

##### 3.2.2 Theme 2: challenges of chatbot-supported decision-making

17 articles were categorized under the theme: Challenges of Chatbot-supported decision-making. The selected articles highlight the drawbacks implementing chatbots into decision-making processes can have. A significant amount of the literature examined highlighted the challenges they had with implementing the technology, with the most prominent issue being the technology’s inaccurate and inconsistent output (Ayoub et al., 2023; Cloux and Monticolo, 2023; Dronkers et al., 2024; Saban and Dubovi, 2024; Huo et al., 2024). Another common challenge is its heavy reliance on high-quality text-based input data, which was pointed out by Garcia Valencia et al. (2023), Gomes (2024), Reicherts et al. (2022), Hsu et al. (2023), Deshpande et al. (2024), and Mihalache et al. (2024), which can be problematic because this type of input might not always be available, thus limiting the context in which these chatbots may be used. Other, fewer mentioned issues were outlined by a paper evaluating the multi-criteria decision-making capabilities of the technology by Wang and Wu (2024), which noted the subpar performance of the technology when compared to human expert decision-making in specific contexts such as supplier selection. This notion was supported by Manivannan et al. (2023), who noted the similar limitation in their paper comparing human to AI decision-making in case-based scenarios. Lastly, its limited language range was critiqued by Lakkaraju et al. (2023) in their evaluation of various chatbots as financial advisors, and they found that some struggled with certain languages and dialects outside of standard English. This evaluation was mirrored by Perez-Soler et al. (2018) in an academic and team decision-making context.

###### 3.2.2.1 Exemplar study 1

This exemplar by Huo et al. (2024) evaluated the performance of large language models: Google Bard, ChatGPT 4, Copilot, and Perplexity in their recommendations and surgical management of Gastroesophageal reflux disease (GERD). Although Google Bard was the most accurate, all the chatbots did not meet the researchers’ expectations when it came to answering clinical questions correctly. Furthermore, they did not answer both patient and practitioner questions with consistent accuracy, and they did not justify their recommendation with respect to established medical guidelines stipulated by the Society of American Gastrointestinal and Endoscopic Surgeons (SAGES). For example, for adults with GERD the chatbots chatbots certainty of the evidence was appropriately provided for 57.1% Key Questions (KQ) by ChatGPT 4.0, 28.6% KQs by Copilot, 42.9% KQs by Google Bard, and 0.0% KQs by Perplexity. The shortcomings are indicative of how these chatbots are not trained for medical applications, severely limiting their ability to be involved in the decision-making processes in this field and could negatively impact overall patient care. Huo et al. (2024) also point out that the tendency for LLMs to hallucinate and produce wordy but incorrect information needs to be addressed before it can be incorporated into medical decision-making or the field in any capacity.

###### 3.2.2.2 Exemplar study 2

Another exemplar by Lakkaraju et al. (2023) encapsulated a lot of the issues that were mentioned by other studies, namely inaccuracies, language limitations, mathematical errors, and perceptual difficulties. This paper presented 13 queries representing banking products such as certificates of deposit and credit cards as well as banking processes such as high value purchases and investment advice. The chatbots used to respond to the queries were Google Bard and Chatgpt and the paper found inconsistencies in the accuracy and personalization of their responses. They did not use all the data they had at their disposal to make personalized decisions and had accurate scores of 53 and 46.17%, respectively. They also tended to make mathematical errors (15.38 and 7.69% respectively) and had mixed results when it came to their abilities to understand other languages besides English. Bard did well with African American Vernacular English but struggled with Telugu (an Indian language), and on the other hand, ChatGPT understood Telugu, but its responses still contained grammatical errors.

###### 3.2.2.3 Research implications

The studies in this theme outline critical areas that then need to be further explored to increase the applicability of technology in the decision-making process. AI chatbots have been demonstrated to be unable to fully match the decision-making capabilities of human experts and often are inaccurate and have inconsistent output; thus, future research is required to get it on par with human experts and justify its incorporation in human decision-making (Wang and Wu, 2024; Gomes, 2024). Huo et al. (2024) and Sarangi et al. (2024) suggest that developing specific chatbots or LLMs that are trained with medical data for medical decisions would enhance the accuracy of the chatbots decisions in the medical context. This suggestion was mirrored by Manivannan et al. (2023), who suggested future work should include enhancing chatbots’ ability to simulate human emotional responses to enhance their accuracy in case-based scenarios by improving their contextual understanding. Several authors who noted the technology’s language limitation suggested that future works focus on improving the chatbot’s ability (its natural language processing) to support multiple languages and dialects, which would make it accessible to a wider range of populations and contexts (Deshpande et al., 2024; Lakkaraju et al., 2023; Perez-Soler et al., 2018). Lastly, the development of systems to train and improve AI chatbots ability to process other forms of input data, such as images, to increase the scope at which the technology can be applied (Mihalache et al., 2024).

###### 3.2.2.4 Practice implications

Articles in this theme outline some valid and significant issues that could arise when utilizing chatbots in the decision-making process. The prevalence of reports of inaccuracies and inconsistency in the current literature strongly suggests that when using chatbots to make decisions, there ought to be human oversight to evaluate the output, and they should not be solely relied on for decision-making (Dronkers et al., 2024; Ayoub et al., 2023; Bužančić et al., 2023). Furthermore, given the technology’s reliance on high-quality text-based input data, it is critical to use clean and extensive input text data to achieve high-quality decision-making from the chatbots (Deshpande et al., 2024). Lastly, some of the literature discussed AI chatbots’ analytical and logical approach, which makes them less susceptible to emotional and situational factors in decision-making, and how this strength could be used in tandem with human decision-making for balanced and improved overall decision-making (Huo et al., 2024; Manivannan et al., 2023).

##### 3.2.3 Theme 3: ethical consideration of using chatbot-supported decision-making

Seven articles were categorized under this theme: ethical considerations of using Chatbot supported decision-making. This theme describes the concerns that were expressed through the literature about the ethics of involving the use of personal data by the chatbot to produce personalized answers and the need for rules and regulations to ensure the use of chatbots is ethical (Garcia Valencia et al., 2023; Karyotaki et al., 2022; Kumar and Joshi, 2022; Leschanowsky et al., 2023; Palade and Ion, 2022; Ramjattan et al., 2021; Sharman, 2024).

###### 3.2.3.1 Exemplar study 1

This exemplar by Sharman (2024) was chosen because it illustrates the significance that AI-driven analysis and decisions can have on users’/consumers’ ability to make independent decisions on significant matters such as policies. This study uses the Cambridge Analytica scandal as a case study to outline how AI-powered data analysis and targeted advertising were used to manipulate individuals’ choices and political opinions, highlighting AI’s potential to erode free will in real-world scenarios. The author concludes that while AI advancements offer numerous benefits, it’s crucial to critically examine their impact on human autonomy and develop ethical frameworks and safeguards to ensure AI’s responsible development and deployment.

###### 3.2.3.2 Exemplar study 2

Leschanowsky et al.’s (2023) study demonstrates the concerns they have about how chatbots get information from users and what they do with that information. A secondary element that was highlighted by the study was the lack of awareness some participants had about data privacy; they were observed sharing information with the chatbot that they normally would have kept to themselves. The study also revealed concerns about the potential for chatbots to manipulate users into disclosing more information than intended, highlighting the importance of having a more user-centric approach prioritizing individual privacy and developing trust between users and AI systems. The researchers identified specific privacy concerns related to monitoring and the potential for secondary use of shared information, which underscores the importance of implementing Privacy by Design principles to ensure that user data is handled responsibly and ethically.

###### 3.2.3.3 Research implications

Given a significant number of researchers noted ethical concerns with the chatbots use of data across various fields, researchers have called for future works to focus on addressing these data concerns. Various approaches have been brought forth, with Kumar and Joshi (2022) suggesting that future work should prioritize developing and strengthening data security and privacy frameworks to ensure responsible AI usage, exploring techniques such as federated learning and differential privacy, and Sharman (2024) believing in the development of systems that focus on human autonomy through their empowerment over their digital interactions and choices. Future research should move beyond artificial research settings and observe user interactions with AI chatbots in real-world environments to gain a more realistic understanding of user behavior and privacy concerns. Studies should be conducted across a broader geographical scope, including participants from different countries and various levels of technological literacy, to examine how cultural norms and privacy regulations influence user perceptions and behaviors. Finally, future work should investigate the “black box” of AI algorithms, exploring the technical aspects of how AI models process and store user data, to identify potential privacy risks and develop effective mitigation strategies (Leschanowsky et al., 2023).

###### 3.2.3.4 Practice implementations

Chatbots require a significant amount of personal data to make personal recommendations; however, in certain fields, such as finances, the medical field, and the educational field, it is important the users consider the security risk of giving chatbots sensitive information (Leschanowsky et al., 2023; Sharman, 2024; Ramjattan et al., 2021). Further, two studies highlighted chatbots ability to manipulate or push users towards a certain decision instead of letting them decide freely; therefore, users should exercise caution about this possibility when using chatbots to make decisions (Sharman, 2024; Reicherts et al., 2022).

### 3.3 Situating ethical concerns within AI governance frameworks

While the reviewed studies highlight concerns such as data privacy, manipulation, transparency, and the need for regulation, these issues align closely with broader AI ethics principles articulated in established governance frameworks. Core principles commonly emphasized in AI ethics literature include fairness, accountability, transparency, explainability, privacy protection, and human oversight. In the context of chatbot-supported decision-making, fairness concerns arise when training data embed historical biases that may influence recommendations. Accountability becomes particularly salient in high-stakes domains such as healthcare and finance, where decision outcomes may directly affect well-being or financial stability. Transparency and explainability are critical given the “black-box” nature of large language models, which may produce outputs without providing traceable reasoning aligned with domain guidelines.

Governance models increasingly emphasize the importance of human-in-the-loop systems, particularly in high-risk applications. The reviewed literature’s frequent recommendation for human oversight aligns with this principle, reinforcing that chatbot systems should augment rather than replace expert decision-makers. Furthermore, emerging regulatory developments globally are moving toward risk-based AI governance, where high-stakes applications require stricter validation, documentation, and monitoring mechanisms. The reliance of many studies on vignette-based testing highlights the need for stronger validation standards before deployment in regulated sectors. Situating chatbot-supported decision-making within these broader ethical and governance frameworks strengthens the argument that ethical considerations are not peripheral constraints but foundational design requirements.

#### 3.3.1 Theme 4: algorithms/tools used in designing chatbots

This theme includes eight articles that all examine the algorithms and various chatbots that were tested in the selected papers. More specifically, they examine the effectiveness of the decision tree decision-making method compared to other methods and compare how chatbots such as Google Bard, ChatGPT 3.5–4.0, Copilot, and Perplexity performed in decision-making in various fields (Deshpande et al., 2024; Gomes, 2024; Hsu et al., 2023; Manivannan et al., 2023; Levkovich and Elyoseph, 2023; Saban and Dubovi, 2024; Sarangi et al., 2024; Wang and Wu, 2024).

##### 3.3.1.1 Exemplar study 1

This exemplar was chosen because it outlined the data, and the algorithms were trained on and compared the accuracy of the algorithms career choice recommendations for the students. The system, which used an aptitude, interest, and personality test, on machine learning models such as the decision tree algorithm, KNN, Adaboost classifier, and Naive Bayes to generate career recommendations, found that decision tree demonstrated the most accurate recommendations with an accuracy of 0.95 consequently, this high accuracy demonstrates the decision tree algorithm’s ability to act as a virtual counsellor through an AI-driven chatbot (Deshpande et al., 2024).

##### 3.3.1.2 Exemplar study 2

The researchers evaluated how well four large language models (LLMs)—Bing, Claude, ChatGPT, and Perplexity— could determine the most appropriate imaging modality based on clinical scenarios, in accordance with the American College of Radiology (ACR) Appropriateness Criteria. The study found that Perplexity achieved the highest accuracy in open-ended (OE) questions, while Bing performed best in select-all-that-apply (SATA) questions. Overall, the scores for OE questions were higher than those for SATA questions. However, there was poor agreement among radiologists in scoring OE responses and strong agreement in scoring SATA responses. The varying performance of LLMs in open-ended and select-all-that-apply questions, as well as the discrepancies in responses observed among different radiologists, underscores the importance of additional fine-tuning and judicious selection by radiologists to ensure consistent and dependable decision support. The findings indicate that the potential of LLMs as adjuncts in healthcare, particularly in radiology, is significant but requires more research and development to ensure their effectiveness, ethical integration, and consistent results.

##### 3.3.1.3 Research implications

The future research should explore expanding the application of ChatGPT-based multi-criteria decion-making methods to a wider range of assessment tasks, such as efficacy in areas like project selection, risk assessment, or investment decision (Wang and Wu, 2024) Furthermore, researchers suggest aligning future research with training LLM chatbots with a wider data set, industry-specific data, and developing standardized testing (Deshpande et al., 2024; Gomes, 2024; Wang and Wu, 2024). Several papers suggest that research on LLM chatbots needs to expand to explore more diverse situations to be able to generalize its use and expand its applicability across different industries (Sarangi et al., 2024; Levkovich and Elyoseph, 2023; Wang and Wu, 2024; Gomes, 2024; Hsu et al., 2023). Since chatbots are better suited for structured learning content, researchers suggest developing chatbots for less structured or open-ended learning domains, which may pose a significant challenge (Hsu et al., 2023).

##### 3.3.1.4 Practice implications

Although the more fine-tuned LLMs that require a subscription to access are generally more accurate than the untrained free versions, Bing still performed with a high degree of accuracy with respect to clinical decision-making (Sarangi et al., 2024).

#### 3.3.2 Theme 5: human vs. AI decision-making

We categorized five articles under the theme of Human vs. AI decision-making. The selected articles focus on comparing the performance of human decision-making and AI decision-making, highlighting the benefits of using one over the other in different contexts. On one hand, AI-driven chatbots demonstrate their superiority to human decision-making due to their lack of susceptibility to bias like humans and high rates of intelligence decay; conversely, they were inferior in overall accuracy in decision-making compared to human expert performance (Banerjee et al., 2017; Gomes, 2024; Levkovich and Elyoseph, 2023; Manivannan et al., 2023; Wang and Wu, 2024).

##### 3.3.2.1 Exemplar study 1

The exemplar by Banerjee et al. (2017) was chosen because it directly compared human and AI-driven decision-making by assessing their performance in specific factors that influence the decision-making process. Human intelligence was found to be better than AI due to humans’ ability to analyze problems using natural perception, psychology, and experience. However, AI-driven chatbots can improve their intelligence through procedural programming and reinforcement. Additionally, since humans have a higher rate of intelligence decay than AI and experience a decline in their human decision-making abilities when experiencing shock or trauma, authors suggest the strengths of AI, namely a lower rate of intelligence decay, can be leveraged to create systems that help humans make better decisions in situations where their judgement is compromised, i.e., after a traumatic experience.

##### 3.3.2.2 Exemplar study 2

Another exemplar, Gomes (2024) explored whether software experts and LLMs made the same assumptions when deciding on which test to execute when conducting a software test. The study compared 127 software engineers who specialized in quality assurance and LLM chatbots ChatGPT 3.5 and 4.0, Google Bard, and Copilot. When presented with a scenario where they had to choose two out of three test cases, 96% of the 127 human participants opted for combinations that offered a wider range of test coverage. In addition, ChatGPT 4.0 and Copilot had a similar preference to human participants for diverse test scenarios, citing reasons like coverage of major functionalities and strategic prioritization. However, the other two chatbots, ChatGPT 3.5 and Bard, consistently recommended test cases focusing on similar scenarios, a choice made by only 3.9% of human testers. Despite the differences in their test case selections, all four chatbots demonstrated an understanding of key testing concepts, such as test diversity, system familiarity, and efficient time management, often mirroring the reasoning provided by human testers. This suggests that chatbots are showing promising signs of being able to assist humans in making informed decisions, potentially leading to more effective software testing practices in the future.

##### 3.3.2.3 Research implications

Future research should investigate the scope of AI chatbot decision-making capabilities and the impact of AI assistance on more realistic testing situations that involve larger and more diverse test suites (Gomes, 2024; Wang and Wu, 2024). Furthermore, Gomes (2024) also recommends aligning future research with the SocraTest taxonomy to better understand how different levels of AI assistance might impact human-AI collaboration in testing. Lastly, to improve decision-making, future research should explore possible techniques to fine-tune the model on industry-specific data, incorporate domain-specific knowledge, or develop more advanced prompting strategies that would help bridge the gap between ChatGPT’s performance and human experts (Wang and Wu, 2024).

##### 3.3.2.4 Practice implications

Some of the selected literature reported that ChatGPT showed promise for decision-making in the medical context and as a multi-criteria decision maker, thus would be the more favorable chatbot to use in those contexts (Levkovich and Elyoseph, 2023; Wang and Wu, 2024). Furthermore, chatbots are able to assess medical situations while avoiding gender or socioeconomic biases that are sometimes observed in primary care physicians, thus enhancing the quality and equity of medical care (Levkovich and Elyoseph, 2023). However, if the data used to train the bot is biased then the bot will reproduce those same biases in its decision-making thus users need to ensure the input data they use is impartial as possible or develop algorithms and classifiers that detect unintentional bias in input (Kumari et al., 2023; Sharman, 2024).

#### 3.3.3 Theme 6: chatbot decision-making in different fields

We categorized 22 articles under the theme of chatbot decision making in different fields. The selected articles investigate the applicability of chatbots as decision-making tools in different contexts, including medical, finance, education, and general group decision-making. For decision-making in the medical field, Sarangi et al. (2024) investigated a wide range of procedures, namely, radiologic decision-making, depression identification, triage, and the development of treatment plans and bilateral vocal folds, among others. With respect to education, the ability to give students career advice and improve their overall wellbeing was explored by Deshpande et al. (2024), Hsu et al. (2023), and Cloux and Monticolo (2023). The utility of chatbots in making financial decisions on both an individual and corporate basis was explored by Azmi et al. (2023), Ferreira et al. (2021), Kumari et al. (2023), Jhaveri et al. (2023), Lakkaraju et al. (2023), Morana et al. (2020), and Temara et al. (2024). Lastly, the enhancement of the team decision-making process when chatbots were included was analyzed by Gurkan and Yan (2023).

##### 3.3.3.1 Exemplar study 1

This exemplar by Huo et al. (2024) was selected due to the study’s exploration of the accuracy of the surgical recommendations chatbots made with respect to the Society of American Gastrointestinal and Endoscopic Surgeons (SAGES) guidelines. In addition, the study compares the accuracy of different LLM chatbots, namely, ChatGPT 4.0, Perplexity, Copilot, and Google Bard. The study concluded that Google Bard was the most accurate LLM in terms of surgical care of gastroesophageal reflux disease (GERD), followed by ChatGPT-4, but none of the chatbots offered right counsel for all clinical queries. The chatbots provided uneven accuracy and responses to both physician and patient inquiries, with performance varying between adult and pediatric situations. Furthermore, the chatbots’ assessments for the confidence of evidence were frequently inaccurate when compared to SAGES guideline ratings, suggesting a significant restriction of their clinical use. This demonstrates that despite their promise, these LLMs need significant improvements to ensure patient safety.

##### 3.3.3.2 Exemplar study 2

The other exemplar by Ramjattan et al. (2021) was chosen because it offered a different perspective on the topic of chatbot decision-making, focusing on the user perception of using chatbots in financial decision-making instead of strictly how the chatbot performs and accuracy. The study investigated a chatbot’s ability to aid users in key financial decision-making areas such as budgeting, spending, saving, and loan affordability. It found that 82% of the participants considered the chatbot highly beneficial for their financial education and decisions. Analysis of open-ended survey responses indicated a strong user desire for chatbots to include features like budget allocation recommendations, investment education, and assistance with spending decisions. These findings suggest that chatbot technology holds promise for promoting financial literacy and positive financial behaviors, particularly among populations vulnerable to debt and financial challenges.

##### 3.3.3.3 Research implications

Several papers indicated that chatbots required very specific text-based data and that they were not trained on medical procedures exclusively (Mihalache et al., 2024; Huo et al., 2024). Consequently, future research needs to focus on the development of more comprehensive guidelines for procedures (as not all have them), because the current lack of standardized protocols for procedures such as Bilateral vocal fold paralysis (BVFP), for example, hinders accurate AI assessment and effective treatment (Dronkers et al., 2024). Several papers reported positive user perceptions in the educational and finance fields; as a result, Morana et al. (2020) suggest that future research should investigate the effect of different social cues on users’ perceived anthropomorphism to establish a more comprehensive framework for designing Robo-Advisor chatbots (Morana et al., 2020; Cloux and Monticolo, 2023; Ramjattan et al., 2021).

##### 3.3.3.4 Practice implications

Several papers expressed concerns about using chatbots in the medical, financial, and educational space, given the user has to input sensitive financial and medical information to get the most accurate feedback from the chatbot; therefore, users need to be cautious of the information they input to the chatbot (Garcia Valencia et al., 2023; Ramjattan et al., 2021; Kumar and Joshi, 2022). Given the inaccuracies technology has demonstrated with its treatment recommendations for patients, Ayoub et al. (2023) stressed that the use of chatbots in clinical decision-making requires human expert oversight, an evaluation supported by Dronkers et al. (2024). Chatbots can be used as an educational tool for delivering personalized financial guidance and promoting positive financial behaviors, particularly among young and low-income adults who may be more susceptible to financial difficulties (Ramjattan et al., 2021).

## 4 Discussion and conclusion

The current body of literature on chatbot-supported decision-making is still emerging, with many studies exploratory in nature. While promising findings exist, particularly in controlled experimental settings, further large-scale, real-world validation studies are needed.

Although six themes were identified in the screening process, several papers explored multiple themes. For example, Kumar and Joshi (2022) focused on the applications of AI in the healthcare sector for enhancement of medical decision making and quality of service, explored the benefits of chatbot decision-making and the ethical considerations that need to be taken in the medical field. In addition, Levkovich and Elyoseph (2023) investigated Human vs. AI decision-making by evaluating ChatGPT 3.5 and 4.0 and highlighting the challenges of chatbot-supported decision-making. Lastly, Manivannan et al.’s (2023) paper also explored Human vs. AI decision-making and weighed the benefits and challenges of chatbot-supported decision-making.

Examining these intersections of the identified themes produces new insights and implications for research and practice. For example, a potential area of exploration could be patient perceptions of chatbot-supported clinical decision-making in their treatment. Such studies could assess human users’ openness to chatbots being used to make decisions pertaining to their health, giving researchers an idea of what aspects of the technology need to be improved to gain the trust of users required to formally implement chatbots in clinical decision-making. Furthermore, studies could explore Human vs. AI decision-making across various disciplinary fields to assess how best to utilize the benefits of using chatbots in decision-making whilst mitigating the limitations of the technology in those respective fields (Gomes, 2024; Manivannan et al., 2023). Furthermore, information from such studies could give further insights into the difference between the two and what aspects of it can be replicated in AI, further improving the decision-making capabilities of the technology (Manivannan et al., 2023).

The descriptive analysis of the articles highlighted areas that require further exploration, namely, increased literature assessing ethical concerns, which would aid with the development of frameworks and guidelines in the coming years to facilitate the implementation of chatbot decision-making in various fields. In addition, the limited disciplinary fields investigated by some articles highlighted an opportunity to expand on the fields chatbot decision-making has been explored in to include engineering, amongst others. Lastly, our analysis suggests that ChatGPT is the most assessed LLM chatbot, suggesting the need for more research into other LLM chatbots such as Google Bard and Perplexity to gain a better understanding of chatbot decision-making by including the strengths and weaknesses of other chatbots. Lastly, several investigations assessed custom-made chatbots developed using decision-making technology and NLPs such as decision trees and google dialog, highlighting the need for more research into the decision-making performance of custom-made chatbots vs. established LLM chatbots.

### 4.1 Implications of methodological dominance of vignette-based studies

A notable methodological pattern across the reviewed literature is the dominance of hypothetical scenarios, vignettes, and case-based evaluations. While these designs allow researchers to systematically compare chatbot outputs under controlled conditions, they may limit ecological validity. Real-world decision-making, particularly in high-stakes domains such as healthcare and finance, often involves incomplete information, contextual uncertainty, time constraints, emotional factors, institutional policies, and ethical considerations that cannot be fully captured through simulated scenarios.

As a result, findings demonstrating chatbot alignment with guidelines or high accuracy in structured case settings should be interpreted with caution. Controlled evaluations may overestimate chatbot performance relative to real-world implementation environments. The current literature therefore reflects an emerging field still largely in experimental or exploratory phases. Future research should prioritize real-world deployment studies, longitudinal evaluations, and externally validated performance assessments to better establish generalizability in high-stakes contexts.

### 4.2 Integrative thematic clarification

While the six themes were analytically categorized, certain conceptual intersections emerged during synthesis. In particular, the themes “Theme 2: Challenges of Chatbot-Supported Decision-Making” and “Theme 5: Human vs. AI Decision-Making” share conceptual proximity. However, they differ in analytical focus. The “Challenges” theme captures intrinsic technological limitations of chatbot systems, including inaccuracies, inconsistencies, hallucinations, mathematical errors, and heavy reliance on high-quality input data. These limitations exist independently of human comparison. In contrast, the “Human vs. AI Decision-Making” theme adopts a comparative evaluative framework, examining how chatbot decision performance aligns with or diverges from human expert judgment, cognitive processes, and susceptibility to bias.

Importantly, comparative studies often amplify the visibility of technological challenges, as discrepancies between chatbot outputs and human expertise reveal performance gaps. Thus, rather than being redundant, these themes operate at different analytical levels: one diagnosing system-level constraints, the other evaluating relative performance within socio-technical contexts. This layered relationship reflects the evolving nature of the field, where chatbot decision-making is simultaneously examined as an independent technological phenomenon and as a collaborator or competitor, within human decision ecosystems.

### 4.3 Conclusion

In conclusion, this SLR features articles published between 2014 and 2024 on chatbots used for decision-making. We retrieved 365 articles from seven databases using relevant search terms and synthesized 36 of them, identifying key themes and publication trends. From these articles, we identified six major themes related to decision-making using chatbots (i) benefits of personalized recommendations in decision-making, (ii) issues with inconsistency in output, (iii) ethical concerns about chatbots using sensitive information to make decisions, (iv) ChatGPT’s decision-making is the most studied, and (v) Human vs. AI Decision-making. The practical and research implications of these findings are further explained in the paper. We also identified future research directions associated with each theme, intended to deepen understanding of using chatbots for decision-making and its possible implications. Through trend analysis, we observed ongoing interest in areas such as the different algorithms/tools that can be used to make chatbots for decision-making as well as the challenges that come with integrating chatbots into a decision-making process. Our findings suggest a continued need for exploration of the ethics of using chatbots’ decision-making across different disciplines, focusing on understanding user perceptions of chatbot integration into decision-making and the development of guideline frameworks that outline ethical approaches to implementing chatbots into the decision-making process. Chatbots demonstrate promising potential across domains, however, the current evidence base remains largely experimental and requires further real-world validation. Furthermore, ethical integration must move beyond reactive safeguards toward proactive governance-by-design approaches in chatbot-mediated decision systems. Finally, our findings highlight the need for a deeper exploration of Human vs. AI decision-making, more specifically how they differ from one another and if and how chatbots can be used to mitigate the shortcomings of human decision-making.

## Data Availability

Publicly available datasets were analyzed in this study. This data can be found here: the articles used in this systematic review are available online.
