# Supplementary material for: Leveraging chatbots for enhanced decision-making: a comprehensive literature review
Source: Front Artif Intell. 2026 Apr 7;9:1748544. doi: 10.3389/frai.2026.1748544 (PMC13095736; doi:10.3389/frai.2026.1748544)
Supplement: Supplementary file 1 [file Data_Sheet_1.docx]

**Appendices**

**Appendix A. Codes, Code Description, and Exemplars**

| **Code** | **Description** | **Exemplar** |
| --- | --- | --- |
| User friendly | Topics describing chatbots used as easy or participants reporting efficiency, quick responses, trust in the decision-making of the chatbots, etc. | When asked if they would use such a chatbot, 75% said yes, 21 % maybe and 6% (Ramjattan et al. 2021) |
| Personalized feedback | Topics describing the utility of the personalized feedback chatbots provide. | By integrating the chatbot with Clinical Decision Support System (CDSS)s, personalized treatment recommendations can be generated based on a patient’s specific characteristics and clinical context (Garcia Valencia et al., 2023) |
| Data centric | Topic centered around the logical and data centric decision-making chatbots | Where most of the decisions were taken by superiors based on their customer knowledge, to now where a tailor-made solution is provided based on data that is read by technology to understand the customer trends. This is what helps in better strategic decision-making and utilizing the resources to the fullest. (Jhaveri et al., 2023) |
| Improved decision outcomes | Studies focused on the improved decision outcomes | Teams supported by the chatbot during the first half of the discussion exhibited higher overall cognitive diversity (M = 0.53, SD = 0.13) than those assisted during the second half (M = 0.43, SD = 0.09). (Gurkan & Yan, 2023) |
| Highly dependent on input data | Topics that highlight the challenges with using a chatbot highly dependent on high-quality input data, the wording of texted based data | The accuracy of the recommendations provided by chatbots depends heavily on the quality and accuracy of the input data. (Garcia Valencia et al., 2023) |
| Inconsistencies and inaccuracies | Topics discussing the inconsistent and inaccurate output chatbot can produce. It covers articles that note the false outputs, hallucinations | The accuracy of both Chatbots was very low regarding their treatment proposals, with a maximum AIPI treatment score in 5% of cases. (Dronkers et al., 2024) |
| Limitations in emotional comprehension | Topics discussing the limitations chatbots have in decision-making due to their limited emotional comprehension | Thirdly, from an information point of view, because technology is still lacking the emotional comprehension, such programs might offer biased personalized information and lack in empathy Palade & Ion (2022). |
| Wordy | Topics that highlighted the extensive responses received from chatbots that were deemed unnecessary by the researchers when testing their decision-making capability | GPT demonstrated its eloquence by occasionally providing information beyond the scope of the prompt; nevertheless, the practical utility of such elaboration remains questionable. (Bužančić et al., 2023) |
| requires oversight | Topics discussing the need for human oversight and review of decisions made by or using chatbots | We believe that even after the improvement of chatbots and their optimization for clinical practice, it will be necessary for HCPs to evaluate AI recommendations and critically make the final decision about the patient (Bužančić et al., 2023) |
| Data privacy concerns | Topics related to the concerns researchers had about the privacy of users’ data in various disciplinary fields | Given the nature of patient health information in kidney transplant care, it is extremely important to have strong encryption methods for securing data at rest and during transmission. (Garcia Valencia et al., 2023 |
| Ethical and privacy regulations and guidelines +transparency | Topics related to the need for regulations of chatbots when they are used to making decisions and the need for transparency with the user on how their information will be utilized | The integration of the chatbot in kidney transplant care calls for clear regulatory frameworks and standardized guidelines. Regulatory authorities and professional organizations should collaborate to establish ethical and legal frameworks that govern the use of AI in healthcare. The standardization of protocols, data formats, and interoperability are essential to facilitate the seamless integration of the chatbot into existing healthcare systems. (Garcia Valencia et al., 2023) |
| Manipulation of user for information | Topics that highlighted the possibility of chatbots manipulating users to share information they otherwise wouldn't | AI-powered algorithms facilitated covert social engineering tactics aimed at manipulating individuals’ social interactions and relationships to influence their beliefs, preferences, and behaviors without their explicit knowledge or consent. By exploiting cultural norms and societal pressures, manipulative campaigns coerced individuals into conforming to predetermined behavioral patterns, further diminishing their autonomy in decision-making processes Shaman, (2024) |
| Decision tree | Topics that discussed the quality of decisions made by the decision tree algorithm on its own or compared to others | Notably, the researcher’s observations reveal varying accuracy scores among different algorithms, with the decision tree classifier showing the highest accuracy. (Deshpande et al., 2024) |
| Custom-made chatbots | Topics that detailed the design of the chatbots that were used to assess the decision-making quality of chatbots | The DialogFlow API was integrated into the Intranet API...obtaining a functional chatbot that allows the execution of operations that assist the management of companies. (Ferreira et al., 2021) |
| Perplexity for open-ended questions | Topics that point out the utility of perplexity when making decisions from open-ended questions compared to others | The accuracy level in the OE question was the highest in Perplexity (0.83) (Sarangi, P. K. et al., 2024) |
| ChatGPT in clinical decision-making | Topics that discussed the utility of chatbots in clinical decision making such as making diagnosis, coming up with treatment plans and triage | This study showed relatively high scores for ChatGPT’s responses to a variety of medical scenarios, and this was especially true for the differential diagnosis and initial treatment plan, but least so for the completeness of the differential diagnosis. Overall, this study shows that ChatGPT could potentially augment clinicians in their daily decision-making but cannot replace a clinician. (Ayoub et al., 2023) |
| ChatGPT can be used as a Multi-Criteria Decision Maker | Topics related to the potential for chatbots to be used in multi-criteria decision making | Furthermore, the table underscores the potential of integrating auxiliary techniques with the base ChatGPT model to enhance its efficacy in the MCDM context (Wang & Wu, 2024) |
| Not susceptible to bias like humans | Topics that refer to chatbots in susceptibility to gender and socioeconomic biases that might influence human decision making | Unlike the treatments proposed by primary care physicians, ChatGPT’s therapeutic recommendations are not tainted by gender or socioeconomic status biases (Levkovich & Elyoseph, 2023) |
| High rates of intelligence decay | Topics that refer to AI's ability to learn increases with experience human | The intelligence decay rate in humans is higher than in an AI. (Banerjee et al., 2017) |
| Did not match human expert performance | Topics that refer to the subpar decision making of chatbots as compared to human experts or their inability to replace humans in their respective fields | It’s also noteworthy that while all configurations of the ChatGPT model exhibit commendable performance, there remains a gap between the best configuration and the ideal human expert performance. (Wang & Wu, 2024) |
| Make decisions by the book | Topics that highlighted the ability for chatbots to make decisions in line with the stipulated guidelines in the medical field | The results of the current study showed that the therapeutic proposals of ChatGPT are in line with the accepted guidelines for mild and severe MD treatment. (Levkovich & Elyoseph, 2023) |
| Group decision-making | Topics that discuss how the use of chatbots in group decision making | It was concluded that the decision-guided chatbot group significantly outperformed the conventional technology-assisted learning group in terms of learning achievements, extrinsic motivation, collective efficacy, cognitive engagement, emotional engagement, and satisfaction with the learning approach. (Aciang Iku-Silan et al., 2023) |
| Financial decision-making | Topics that discuss the potential for chatbots to be used in personal finances, corporate and managerial decision making | We believe these results show that the sample of our target groups has a strong inclination towards this type of approach to improving their financial education and behavior. (Ramjattan et al., 2021) |
| Mathematical errors | Topics that point out the limitations of chatbots when it comes to executing mathematical calculations in a medical context | 7.69% of ChatGPT Queries had mathematical errors. 15.38% of Bard queries had mathematical errors. (Lakkaraju et al., 2023) |
| Guiding student career choice | Topics related to chatbots being used as academic and career guidance and overall wellbeing for high school students | A key feature is the integration of an AI chatbot, acting as a virtual counselor to provide real-time, personalized responses. It suggests the best careers based on their interests and skills. The system utilizes a decision tree classifier, conducting tests to recommend the best career path for students based on their interests and aptitudes (Deshpande et al., 2024). |

**Appendix B: Classification of the Reviewed Studies Based on Specific Themes**

**Theme 1: Benefits of using chatbot for decision-making**

| **Author** | **Country of affiliation** | **Title** |
| --- | --- | --- |
| Ingrams et al., 2021 | The Netherlands | In AI we trust? Citizen perceptions of AI in government decision making |
| Reicherts et al., 2022 | UK | "Extending Chatbots to Probe Users: Enhancing Complex Decision-Making Through Probing Conversations." |
| Ferreira et al., 2021 | Portugal | "A Step Towards the Use of Chatbots to Support the Enterprise Decision-Making Processes" |
| Perez-Soler et al., 2018 | Spain | Collaborative Modeling and Group Decision Making Using Chatbots in Social Networks |
| Azmi et al., 2023 | Morrocco | A Context-Aware Empowering Business with AI: Case of Chatbots in Business Intelligence Systems |
| Cloux & Monticolo, 2023 | France | High School Students' Career Decision-Making Process: Impact Assessment of a Computer-assisted Career Guidance System |
| Ramjattan et al., 2021 | Trinidad and Tobago | Using Chatbot Technologies to help Individuals make Sound Personalized Financial Decisions |
| Morana et al., 2020 | Morocco | The Effect of Anthropomorphism on Investment Decision-Making with Robo-Advisor Chatbots |
| Aciang Iku-Silan et al., 2023 | Taiwan | Decision-guided chatbots and cognitive styles in interdisciplinary learning |
| Rodriguez‐Arrastia et al., 2022 | Spain | Experiences and perceptions of final-year nursing students of using a chatbot in a simulated emergency situation: A qualitative study |
| Kumar & Joshi, 2022 | India | Applications of AI in Healthcare Sector for Enhancement of Medical Decision Making and Quality of Service |
| Garcia Valencia et al., 2023 | USA | Enhancing Kidney Transplant Care through the Integration of Chatbot |
| Karyotaki et al., 2022 | Greece | Chatbots as Cognitive, Educational, Advisory & Coaching Systems |
| Sheetal Temara et al., 2024 | USA | Using AI and Natural Language Processing to Enhance Consumer Banking Decision-Making |
| Jhaveri et al., 2023 | India | The Efficacy of Artificial Intelligence in making Best Marketing Decisions |
| Alaaeldin et al., 2021 | Egypt | DEVELOPING CHATBOT SYSTEM TO SUPPORT DECISION MAKING BASED ON BIG DATA ANALYTICS |
| Manivannan, K. et al., 2023 | India | The Deciding Divide: A Comparative Study on Exploring the Parallels and Contrasts in Decision-Making between Humans and Artificial Intelligence |
| Gurkan & Yan, 2023 | USA | Early Chatbot Assistance Can Enhance Team Decision-Making by Promoting Cognitive Diversity and Information Elaboration |
| Banerjee et al., 2017 | India | A comparative study on decision-making capability between human and artificial intelligence |
| **Theme 2: Challenges of Chatbot-supported decision-making** | | |
| **Author** | **Country of affiliation** | **Title** |
| Ayoub et al. 2023 | USA | Mind + Machine: ChatGPT as a Basic Clinical Decisions Support Tool |
| Cloux and Monticolo 2023 | France | High School Students' Career Decision-Making Process: Impact Assessment of a Computer-assisted Career Guidance System |
| Deshpande et al. 2024 | Nepal | Implementation of an NLP-Driven Chatbot and ML Algorithms for Career Counseling |
| Dronkers et al. 2024 | UK | Evaluating the Potential of AI Chatbots in Treatment Decision-making for Acquired Bilateral Vocal Fold Paralysis in Adults. |
| Garcia Valencia et al. 2023 | USA | Enhancing Kidney Transplant Care through the Integration of Chatbot |
| Gomes 2024 | Sweden | Unveiling Assumptions: Exploring the Decisions of AI Chatbots and Human Testers |
| Hsu et al. 2023 | Taiwan | Effects of Incorporating an Expert Decision-Making Mechanism into Chatbots on Students' Achievement, Enjoyment, and Anxiety |
| Huo et al. 2024 | Canada | The performance of artificial intelligence large language model-linked chatbots in surgical decision-making for gastroesophageal reflux disease |
| Ingrams et al. 2021 | the Netherlands | In AI we trust? Citizen perceptions of AI in government decision making |
| Lakkaraju et al. 2023 | United States | Can LLMs be Good Financial Advisors? An Initial Study in Personal Decision Making for Optimized Outcomes |
| Manivannan et al. 2023 | India | The Deciding Divide: A Comparative Study on Exploring the Parallels and Contrasts in Decision-Making between Humans and Artificial Intelligence |
| Mihalache et al. 2024 | Canada | Interpretation of Clinical Retinal Images Using an Artificial Intelligence Chatbot |
| Perez-Soler et al. 2018 | Spain | Collaborative Modeling and Group Decision Making Using Chatbots in Social Networks |
| Reicherts et al. 2022 | UK | Extending Chatbots to Probe Users: Enhancing Complex Decision-Making Through Probing Conversations. |
| Sarangi, P. K. et al. 2024 | India | Radiologic Decision-Making for Imaging in Pulmonary Embolism: Accuracy and Reliability of Large Language Models—Bing, Claude, ChatGPT, and Perplexity |
| Saban and Dubovi 2024 | Israel | A comparative vignette study: evaluating the potential role of generative AI model in enhancing clinical decision making in nursing |
| Wang and Wu 2024 | China | CAN CHATGPT SERVE AS A MULTI-CRITERIA DECISION MAKER? A NOVEL APPROACH TO SUPPLIER EVALUATION |
| **Theme 3: Ethical consideration of using Chatbot-supported decision-making** | | |
| **Author** | **Country of affiliation** | **Title** |
| Garcia Valencia et al. 2023 | USA | Enhancing Kidney Transplant Care through the Integration of Chatbot |
| Karyotaki et al. 2022 | Greece | Chatbots as Cognitive, Educational, Advisory & Coaching Systems |
| Kumar and Joshi, 2022 | India | Applications of AI in Healthcare Sector for Enhancement of Medical Decision Making and Quality of Service |
| Leschanowsky et al. 2023 | United States | Privacy Strategies for Conversational AI and their Influence on Users' Perceptions and Decision-Making |
| Ramjattan et al. 2021 | Trinidad and Tobago | Using Chatbot Technologies to help Individuals make Sound Personalized Financial Decisions |
| Sharman, 2024 | Norway | The Enigma Unveiled: How AI Compromises Free Will in Decision-Making |
| Palade & Ion, 2022 | Romania | A PRELIMINARY STUDY ON USING CHATBOTS APPLICATIONS TO SUPPORT ARTIFICIAL INTELLIGENCE DECISION MAKING PROCESS |
| **Theme 4: Algorithms/tools used in designing chatbots** | | |
| **Author** | **Country of affiliation** | **Title** |
| Deshpande et al. (2024) | Nepal | Implementation of an NLP-Driven Chatbot and ML Algorithms for Career Counseling |
| Gomes (2024) | Sweden | Unveiling Assumptions: Exploring the Decisions of AI Chatbots and Human Testers |
| Hsu et al. (2023) | Taiwan | Effects of Incorporating an Expert Decision-Making Mechanism into Chatbots on Students' Achievement, Enjoyment, and Anxiety |
| Manivannan, K et al. (2023) | India | The Deciding Divide: A Comparative Study on Exploring the Parallels and Contrasts in Decision-Making between Humans and Artificial Intelligence |
| Levkovich and Elyoseph (2023) | Israel | Identifying depression and its determinants upon initiating treatment: ChatGPT versus primary care physicians |
| Saban and Dubovi (2024) | Israel | A comparative vignette study: evaluating the potential role of generative AI model in enhancing clinical decision making in nursing |
| Sarangi, P. K. et al. (2024) | India | Radiologic Decision-Making for Imaging in Pulmonary Embolism: Accuracy and Reliability of Large Language Models—Bing, Claude, ChatGPT, and Perplexity |
| Wang and Wu (2024) | China | CAN CHATGPT SERVE AS A MULTI-CRITERIA DECISION MAKER? A NOVEL APPROACH TO SUPPLIER EVALUATION |
| **Theme 5: Human vs. AI Decision-making** | | |
| **Author** | **Country of affiliation** | **Title** |
| Banerjee et al. (2017) | India | A comparative study on decision-making capability between human and artificial intelligence |
| Gomes (2024) | Sweden | Unveiling Assumptions: Exploring the Decisions of AI Chatbots and Human Testers |
| Levkovich and Elyoseph (2023) | Israel | Identifying depression and its determinants upon initiating treatment: ChatGPT versus primary care physicians |
| Manivannan, K et al. (2023) | India | The Deciding Divide: A Comparative Study on Exploring the Parallels and Contrasts in Decision-Making between Humans and Artificial Intelligence |
| Wang and Wu (2024) | China | CAN CHATGPT SERVE AS A MULTI-CRITERIA DECISION MAKER? A NOVEL APPROACH TO SUPPLIER EVALUATION |
| **Theme 6: Chatbot decision-making in different fields** | | |
| **Author** | **Country of affiliation** | **Title** |
| Ayoub et al. (2023) | USA | Mind + Machine: ChatGPT as a Basic Clinical Decisions Support Tool |
| Azmi et al. (2023) | Morrocco | A Context-Aware Empowering Business with AI: Case of Chatbots in Business Intelligence Systems |
| Bužančić et al. (2023) | Croatia | Clinical decision-making in benzodiazepine deprescribing by healthcare providers *vs*. AI-assisted approach |
| Cloux and Monticolo (2023) | France | High School Students' Career Decision-Making Process: Impact Assessment of a Computer-assisted Career Guidance System |
| Deshpande et al. (2024) | Nepal | Implementation of an NLP-Driven Chatbot and ML Algorithms for Career Counseling |
| Dronkers et al. (2024) | UK | Evaluating the Potential of AI Chatbots in Treatment Decision-making for Acquired Bilateral Vocal Fold Paralysis in Adults. |
| Ferreira et al. (2021) | Portugal | "A Step Towards the Use of Chatbots to Support the Enterprise Decision-Making Processes" |
| Garcia Valencia et al. (2023) | USA | Enhancing Kidney Transplant Care through the Integration of Chatbot |
| Hsu et al. (2023) | Taiwan | Effects of Incorporating an Expert Decision-Making Mechanism into Chatbots on Students' Achievement, Enjoyment, and Anxiety |
| Huo et al. (2024) | Canada | The performance of artificial intelligence large language model-linked chatbots in surgical decision-making for gastroesophageal reflux disease |
| Jhaveri et al. (2023) | India | The Efficacy of Artificial Intelligence in making Best Marketing Decisions |
| Kumar and Joshi (2022) | India | Applications of AI in Healthcare Sector for Enhancement of Medical Decision Making and Quality of Service |
| Kumari et al. (2023) | India | Artificial Intelligence's Effects on Corporate Decision-Making Processes |
| Lakkaraju et al. (2023) | United States | Can LLMs be Good Financial Advisors?: An Initial Study in Personal Decision Making for Optimized Outcomes |
| Levkovich and Elyoseph (2023) | Israel | Identifying depression and its determinants upon initiating treatment: ChatGPT versus primary care physicians |
| Mihalache et al. (2024) | Canada | Interpretation of Clinical Retinal Images Using an Artificial Intelligence Chatbot |
| Morana et al. (2020) | Morocco | The Effect of Anthropomorphism on Investment Decision-Making with Robo-Advisor Chatbots |
| PALADE and ION (2022) | Romania | A PRELIMINARY STUDY ON USING CHATBOTS APPLICATIONS TO SUPPORT ARTIFICIAL INTELLIGENCE DECISION MAKING PROCESS |
| Sarangi, P. K. et al. (2024) | India | Radiologic Decision-Making for Imaging in Pulmonary Embolism: Accuracy and Reliability of Large Language Models—Bing, Claude, ChatGPT, and Perplexity |
| Ramjattan et al. (2021) | Trinidad and Tobago | Using Chatbot Technologies to help Individuals make Sound Personalized Financial Decisions |
| Reicherts et al. (2022) | UK | Extending Chatbots to Probe Users: Enhancing Complex Decision-Making Through Probing Conversations |
| Rodriguez‐Arrastia et al. (2022) | Spain | Experiences and perceptions of final-year nursing students of using a chatbot in a simulated emergency situation: A qualitative study |
| Saban and Dubovi (2024) | Israel | A comparative vignette study: evaluating the potential role of generative AI model in enhancing clinical decision making in nursing |
| Sheetal Temara et al. (2024) | USA | Using AI and Natural Language Processing to Enhance Consumer Banking Decision-Making |
